# Changes in Eating Habits and Lifestyles in a Peruvian Population during Social Isolation for the COVID-19 Pandemic

**DOI:** 10.1155/2021/4119620

**Published:** 2021-12-01

**Authors:** Salomón Huancahuire-Vega, Edda E. Newball-Noriega, Ricardo Rojas-Humpire, Jacksaint Saintila, Mery Rodriguez-Vásquez, Percy. G. Ruiz-Mamani, Wilter C. Morales-García, Michael White

**Affiliations:** ^1^Grupo de Investigación P53, Escuela de Medicina Humana, Facultad de Ciencias de la Salud, Universidad Peruana Unión, Lima, Peru; ^2^Departamento de Nutrición, Escuela de Nutrición Humana, Facultad de Ciencias de la Salud, Universidad Peruana Unión, Lima, Peru; ^3^Escuela Profesional de Enfermería, Facultad de Ciencias de la Salud, Universidad Privada San Juan Bautista, Lima, Peru; ^4^Escuela de Posgrado, Universidad Peruana Unión, Lima, Peru; ^5^Dirección General de Investigación, Universidad Peruana Unión, Lima, Peru

## Abstract

**Background:**

Peru has one of the highest infection and death rates in the world for the COVID-19 pandemic. The government implemented house confinement measures with probable consequences on lifestyle, particularly affecting eating habits, physical activity, sleep quality, and mental health.

**Objectives:**

The aim of this study was to assess the lifestyles, physical activity, and sleep characteristics, as well as changes in eating habits in a Peruvian population during the first wave of the COVID-19 pandemic.

**Methods:**

A cross-sectional descriptive study was performed. We analyzed Peruvian adults based on an online self-administered questionnaire divided into sociodemographic, anthropometrics, COVID-19 diagnosis reported, lifestyle habits, and frequency of consumption of foods.

**Results:**

During confinement for COVID-19, 1176 participants were studied. Of these, most reported weight gain (1 to 3 kg) and 35.7% were overweight. The lifestyles habits showed that 54.8% reported doing physical activity and 37.2% sleep less. The Peruvian sample presented a main meal pattern of breakfast (95.7%), lunch (97.5%), and dinner (89.1%). Likewise, eating habits before and during COVID-19 pandemic showed that vegetables (OR:1.56, CI95% 1.21–200), fruit (OR: 1.42, CI95% 1.10–1.81), legumes (OR:1.67, CI95% 1.23–2.28), and eggs (OR: 2.00, CI95% 1.52–2.65) presented significant consumption increase during social isolation, while bakery products (OR: 0.74, CI95% 0.56–0.97), meat, snack, refreshment, and fast food decreased in consumption. Other foods showed no significant differences.

**Conclusion:**

This study showed an important frequency of overweight and sleep changes. There was a slight increase in physical activity despite the social isolation measures and an increase in healthy eating habits; nevertheless, the majority reported gaining weight.

## 1. Introduction

An announcement made by the WHO on January 30, 2020, declared COVID-19 a global health emergency due to the exponential growth of cases in China and other countries of the world [1]. As of July 23, 2021, according to the Coronavirus Resources Center at Johns Hopkins University, more than 192.7 million cases have been confirmed, while the number of deaths has risen to more than 4.139 million [2]. Given the circumstances and the measures needed to control the spread of the disease, government authorities were forced to take decisive actions, such as social distancing and mandatory isolation in homes, which have led to restrictions on various daily activities, with probable consequences on lifestyle, particularly affecting eating habits, physical activity, sleep quality, and mental health [3].

Peru is among the countries with the highest global contagion and mortality rates from COVID-19, which has led to estimating that life expectancy in this country has decreased during 2020 by more than two years [4]. In this country, the first case of COVID-19 infection was reported by the government on March 6, 2020. Since then, the government has implemented a series of preventive measures in order to limit and contain the spread of the disease. On March 16, the Peruvian government, through Supreme Decree No. 008-2020-SA, declared a nationwide state of health emergency for a period of ninety (90) calendar days, dictating prevention measures and mandatory social isolation (national quarantine) for the control of COVID-19 [5]. Subsequently, the government announced an extension that modified the restriction to a targeted social isolation, but the state of health emergency and social distancing measures were extended repeatedly and currently are set until September 31, 2021 [6].

The period of social isolation due to COVID-19 has caused a series of changes in people's daily routine and lifestyle [7]. Available evidence has shown that during the pandemic, physical activity decreased in the population, while the amount of screen time increased significantly [7]. In fact, prior to the pandemic, these factors, along with obesity, were described as a public health problem in the Peruvian population, and COVID-19 may make this situation even worse [8]. In addition, studies have shown the negative impact of sedentary behaviors on physical and mental health [9]. Likewise, the fear caused by COVID-19 has an indirect effect, causing negative emotions and affecting the quality of sleep, contributing to an increase in obesity [10].

Previous studies have demonstrated the beneficial effects of physical activity and sleep quality on mental health [11]. In addition, adequate physical activity can improve the quality of sleep, reducing the risk of morbidity from chronic noncommunicable diseases [12]. Furthermore, appropriate eating habits can be a protective factor for physical and mental health, disease prevention, and strengthening of the immune system [13]. Indeed, improved lifestyle, as a possible strategy that includes regular physical activity, less screen time, adequate rest, and proper dietary habits through a diet based on consumption of minimally processed plant foods and healthy fats, would lead to better control of chronic noncommunicable diseases and thus strengthen the immune system during the COVID-19 pandemic [13].

Additionally, evidence has shown that the risks of choosing inappropriate eating behaviors can increase as a psychological and emotional response to COVID-19 [14]. In fact, it has already been shown that negative emotional experiences can lead to overeating [15]. Eating habits characterized by a high consumption of ultra-processed, high-calorie-density foods and low intake of fiber, antioxidants, and other bioactive elements can affect the immune system, causing chronic activation of the innate system and inhibition of the adaptive immune system response by increasing oxidative stress, thus delaying defensive intervention against pathogens [16].

Many studies have evaluated dietary changes during the COVID-19 pandemic in different age groups and countries [3, 17, 18], but, thus far, there is no study among adults from a Peruvian population. The objective of the present study was to evaluate the lifestyles, physical activity levels, and sleep characteristics, as well as changes in eating habits, in a Peruvian population during the period of social isolation due to the first wave of the COVID-19 pandemic.

## 2. Materials and Methods

### 2.1. Design and Participants

This is a cross-sectional descriptive study based on a self-administered questionnaire. This questionnaire was addressed to the Peruvian adult population (over 18 years of age) and was applied using nonprobabilistic convenience sampling. We included participants between 18 and 79 years old of both sexes from the different regions of Peru. Responses from 1218 participants were obtained, of which 42 were excluded because they did not completely fill out the questionnaire or did not sign informed consent, or certain inconsistencies related to age, height, weight, and so on cast suspicion on their accuracy.

The study followed the international ethical recommendations contained in The Declaration of Helsinki. All participants were informed about the characteristics of the study, as well as their anonymous, voluntary, and confidential participation. This study was approved by the Ethics in Research Committee of the Universidad Peruana Unión (No. 2020-CEUPeU-00013).

### 2.2. Procedure

The questionnaire was applied from July 16 to August 31, 2020 (while the Peruvian population was in partial confinement), using the Google Forms platform and disseminated through social networks (Facebook and WhatsApp) and institutional mailing lists. The questionnaire was completed through any electronic device with an Internet connection. On the initial page, the participants were explained the voluntary nature of their participation with informed consent, as well as the justification, objectives, possible risks, and benefits of the study before continuing with the instrument itself.

### 2.3. Questionnaire and Variables

The questionnaire was developed by the authors to specifically address the particularities of the current pandemic context and its impact on lifestyle. The questionnaire included 42 questions divided into four sections: sociodemographic data (10 questions: gender, marital status, age, level of education, occupational status, employment status, if the social isolation mandate was adhered to, duration of social isolation, and people in the home before and during social isolation), anthropometric data and COVID-19 diagnosis reported (5 questions: weight, height, weight change during social isolation, number of kilograms, and COVID-19 diagnosis), information on lifestyle habits (11 questions: physical activity, change and hours of sleep, and meals eaten per day during confinement), information on the frequency of consumption of the main food groups (16 questions: vegetables, fruit, legumes, dried fruit/nuts, meat, fish, eggs, milk, yogurt, bakery products, snacks, beverages, beer, wine, other alcohol, and fast food) before and during social isolation, based on five response categories: participants who reported having consumed food from 1 to 4 times/week were considered as low consumption, while high consumption was considered when they reported having consumed from 5 times/week to 2 or more times/day [19].

### 2.4. Statistical Analysis

The data analysis was performed in RStudio v4.0.2 (R Foundation for Statistical Computing, Vienna, Austria; http://www.R-project.org). The variables were encoded and analyzed as categorical. To descriptive analysis, categorical variables were clustered into tables with absolute and relative frequency (%). For the comparative analysis, *χ*^2^ or Fisher's exact test was used for categorical variables depending on distribution of each variable. Subsequently, to determinate differences before and during the COVID-19 pandemic, the McNemar test was performed. Moreover, logistic regression models were performed to analyze the factors that influenced the odds of BMI and sleep changes. The results of logistic regression analyses were expressed as crude odds ratio (cOR), adjusted odds ratio (aOR) by sex and age, and 95% confidence intervals (95% CI). For all analyses, *p* < 0.05 was considered statistically significant.

## 3. Results

A summary of the sample's sociodemographic characteristics can be found in Table 1. In total, 1176 participants were studied, 571 men and 605 women. The largest age groups were 18 to 25 years old (44.4%) with predominant women and 26 to 40 years old (33.7%) with predominant men. Overall, 67.7% were single and 38% had completed university studies. During social isolation, 39% of those sampled were students, 37.5% were workers, 30.8% worked with a formal contract, 46% were not working, and 67.6% were under social isolation for more than 61 days as of the date of the study.

A total of 35.7% of those studied were overweight and 8.4% were obese, with a majority of men in both groups. A large portion reported having gained weight, 20.8% and 13.7% between 1 to 3 kg and 4 to 7 kg, respectively. Of all respondents, 8.7% had a confirmed diagnosis of COVID-19.

Some aspects of lifestyles showed patterns and changes when grouped by sex during the social isolation (Table 2). More than half of those studied affirmed to doing physical activity (54.8%) between 1 and 2 (14.7%) and 3 to 4 (26.4%) days per week, for at least 30 min. The reported sleep routine presented significate changes, wherein 37.2% responded that they slept less and 36.9% slept more hours at night compared to before the pandemic. The hours of sleep per night were largely between 4 and 6 (35.6%) and 7 to 9 (55.4%) hours.

**Figure 1:**
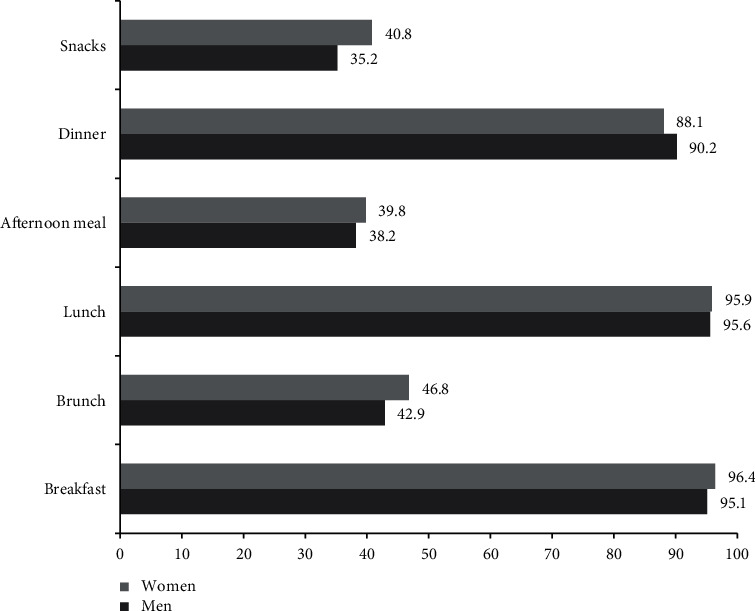
Frequency of eating patterns during social isolation.

Eating patterns during the social isolation showed that approximately 90 to 95% ate breakfast, lunch, and dinner. Additionally, some extra food consumption was reported for brunch (42.9 to 46.8%), afternoon meal (38.2 to 39.8%), and snacks (35.2 to 40.8%); these results presented no differences by sex (Figure 1).

**Figure 2:**
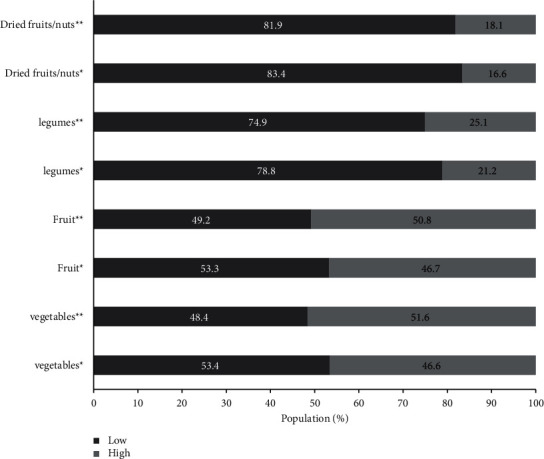
Changes in the frequency of low and high consumption of plant-based foods before and during social isolation. ^*∗*^Before social isolation; ^*∗∗*^during social isolation.

**Figure 3:**
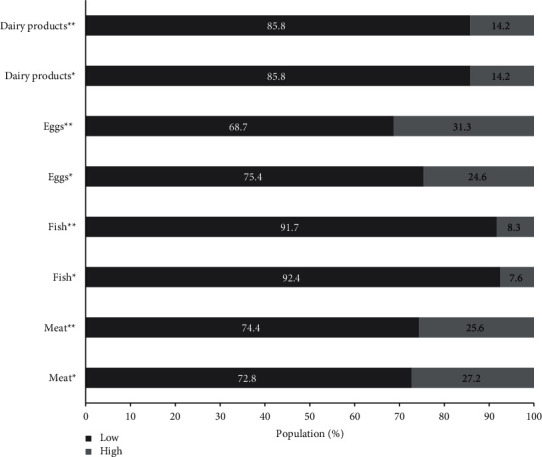
Changes in the frequency of low and high consumption of animal-based foods before and during social isolation. ^*∗*^Before social isolation; ^*∗∗*^during social isolation.

**Figure 4:**
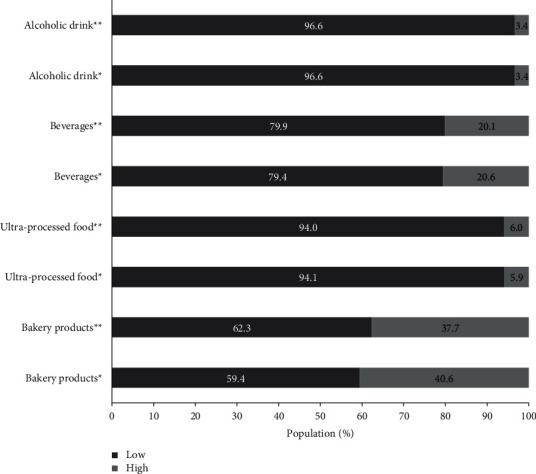
Changes in the frequency of low and high consumption of processed food before and during social isolation. ^*∗*^Before social isolation; ^*∗∗*^during social isolation.

The eating habits showed some changes before and during social isolation (Table 3). Fruit and vegetables presented elevated frequency of consumption, while legumes and dried fruit/nuts showed low frequency of consumption (Figure 2). On the other hand, just vegetables (OR:1.56, CI95% 1.21–200), fruit (OR: 1.42, CI95% 1.10–1.81), and legumes (OR:1.67, CI95% 1.23–2.28) presented significant consumption increase during social isolation. Approximately, a quarter of the population presented high consumption of meat and eggs (Figure 3), while dairy products and fish showed low frequency of consumption. Among the animal-based foods, only eggs (OR: 2.00, CI95% 1.52–2.65) presented significant consumption increase during social isolation. The consumption of alcoholic drinks and ultra-processed food was the lowest, while approximately 20% and 40% of sample presented high consumption of beverages and bakery products, respectively (Figure 4). In the processed food, just bakery products (OR: 0.74, CI95% 0.56–0.97) presented change before and during social isolation.

Additionally, to determine factors associated with BMI and sleep changes, a multivariate regression analysis was made. Some factors showed association with BMI in the study sample during the COVID-19 pandemic (Table 4). In this sense, women (cOR:0.38, CI95%: 0.30–0.48), vegetable consumption (cOR:0.77, CI95%: 0.61–0.97), physical activity (PA) at home (cOR:0.55, CI95%: 0.44–0.70), PA per week (cOR:0.89, CI95%: 0.89–0.94), and time of PA decreased the probability of high BMI in bivariate analysis. In the adjusted analysis, PA at home (aOR:0.58, CI95%: 0.45–0.74) reduced the probability of high BMI by 42%, PA of less than 30 min until 1 hour reduced the probability of high BMI by 39% and 46%, respectively, and the addition of each day of PA reduced the probability of high BMI by 11%. Furthermore, the addition of each additional year of age increases the probability of high BMI by 5%.

The sleep changes (sleep more or less) presented an association with some variables during the COVID-19 pandemic (Table 5). In this sense, women (cOR:1.52, CI95%: 1.17–1.98), social isolation more than 61 days (cOR:2.30, CI95%: 1.57–3.35), and meals per day (cOR:1.21, CI95%: 1.08–1.35) showed an increase of sleep changes in bivariate analysis. In the adjusted analysis, the social isolation for more than 61 days increased the probability of sleep changes by 2.31 times (aOR:2.31, CI95%: 1.57–3.38), while the addition of each additional meal in the day increased the probability of sleep changes by 17% (aOR: 1.17, CI95%: 1.05–1.32). Additionally, the addition of each year of age decreased the probability of sleep changes in this population by 2%.

## 4. Discussion

During social isolation due to the first wave of the COVID-19 pandemic, most Peruvians participants remained constant in some of the key variables under study. Among those who changed, the change was often towards healthier eating habits, with an increase in the frequency of consumption of vegetables, fruit, legumes, eggs, and a decrease in bakery products. More than 70% of the participants reported changes in their sleep pattern, and more than half indicated an increase in physical activity despite the social isolation. However, perception of weight gain was also reported.

This study highlights the potential impact of social isolation due to COVID-19 by including a variety of weight-related behaviors in Peruvian adults. The results of this study indicate that of all the participants subjected to social isolation measures, 35.7% and 8.4% were overweight and obese, respectively. In addition, it is also evidenced that half of the participants gained weight. Likewise, the findings show that the imposition of social isolation influenced lifestyles causing negative effects and weight changes, as the participants reported gaining weight with 20.8% between 1 and 3 kg and 13.7% from 4 to 7 kg, respectively. Similar studies in Ibero-American countries indicated a weight gain in 48.6% of participants during social isolation [20]. Moreover, a study with 639 Chilean participants indicated an average weight gain of 1.99 kg [21]. These similar results may be because the population of this region of the world has similar eating habits, as was demonstrated in a representative sample of the urban population of Latin America [22].

On the other hand, studies in different Latin American countries such as Argentina, Mexico, and Brazil showed high proportions of their population with overweight and obesity during the period of social isolation [20]. Additionally, in another study with Chilean adults, an increase of 0.7 units of BMI was reported [21]. The population of these countries presented a low overall diet quality score and low dietary diversity [23]. Additionally, during the first mandatory isolation, in Peru as in these countries with high prevalence of obesity, low levels of physical activity were reported [24]. These factors could be influencing the increase of BMI.

Although energy intake and energy expenditure were not measured, it is possible that there is a possible imbalance between them, which could be contributing to weight gain, regardless of the quality of the diet. Higher daily energy intakes were reported during social isolation periods due to the pandemic [25], and other factors that could contribute to the weight gain perceived by the participants could be alterations in their sleep pattern [26] and high rates of stress and depression that lead people to consume foods with high sugar content, irregularity of the meals, with a greater consumption of food outside the main meals (snacks and intermediate meals in the morning and afternoon) [27].

It is recommended to adopt and maintain healthy lifestyle habits as a fundamental principle of physical health, especially during confinement due to COVID-19. Prepandemic studies showed that the Peruvian population had a high prevalence of physical inactivity and sedentary behaviors [28]. This study found changes in behavior during social isolation; the majority of participants claimed to be physically active (54.8%) for 1-2 (14.7%) or 3-4 (26.4%) days per week, for at least 30 minutes. Similar studies showed increased reporting of moderate and vigorous physical activity in older adults during social isolation [29]. On the other hand, other studies show that total physical activity decreased significantly during social isolation, due to greater sedentary behavior, more prolonged during the weekend, increase in sitting time, or only low-intensity activities such as housework [30].

This sudden cessation of physical exercise is detrimental in the context of this disease, since this decrease is associated with rapid muscle atrophy and reduced energy consumption by the muscles, promoting obesity and lipid accumulation, decreased ability of organ systems to resist viral infection, and increased risk of damage to the immune system, respiratory system, cardiovascular system, musculoskeletal system, and brain [31]. Additionally, in this study, the physical activity at home, at least 30 minutes per day and more days of practice per week, significantly reduced the probability of high BMI. That is why the WHO recommends at least 150 minutes per week of moderate to vigorous intensity physical activity or 75 minutes of high-intensity physical activity per week or a combination of both [32].

Sleep plays a critical role in physical and mental health, and adequate sleep duration and quality are essential to cope with major life events such as the COVID-19 pandemic [33]. More than 70% of the participants in this study reported substantial changes in their sleep patterns during the period of isolation due to COVID-19. Similar studies revealed a high prevalence of anxiety sleep disorders and depressive symptoms in the population during the COVID-19 social isolation period, with women reporting a higher risk of sleep difficulties [34], which was also observed in our study. It is likely that women were more receptive to expressing psychological distress and somatic symptoms [35]. When the factors associated with alterations in sleep habits were analyzed, it was determined that the social isolation for more than 61 days increased the probability of sleep changes by 2.31 times. On the other hand, the uncertainty of being infected by the coronavirus would cause greater pressure in the population, relating to hypochondriacal concerns, sleep apnea, and childhood trauma that can be reflected in sleep mentality, affecting its quality [36]. Most of the participants in this study were students and workers under the age of 40, who probably had to change their activities from in-person to online, and these changes in their routines could have influenced and altered the physiological regulators of sleep.

This study also showed significant changes in the eating habits that this Peruvian population adopted during social isolation, among which the increase of eggs, legumes, fruit, and vegetables, as well as the decrease of bakery products. Some studies observed an improvement in the pattern of consumption of healthy foods and a restriction of unhealthy foods, especially in the younger population (<30 years) [37]. In contrast, studies in Italy indicated changes in eating habits in almost half the population so that women were more likely than men to increase their food intake to feel better [9]. In other countries, after-dinner snacks, carbohydrate consumption, consumption of sweets, cookies, and cakes and consumption of fresh food products increased [26]. The lack of dietary and eating restriction may be due to a response to stress and reduced physical activity, which could lead to decreased nutritional quality [26]. Additionally, the addition of each extra meal during the day increased the probability of sleep changes by 17%. This unhealthy behavior is observed in other studies, primarily in adult populations with obesity and in settings with a lower eating environment index [38].

Prepandemic studies carried out in the Latin American population, including Peru, revealed low dietary diversity and low frequency of consumption of food groups rich in micronutrients, so they deserve a lot of attention and care [23]. Although the Peruvian population had the highest consumption of fruit and whole grains and the lowest consumption of red and processed meats compared to other Latin American countries, they had the highest consumption of homemade sugar-sweetened beverages [22]. In our study, 80% of the participants indicated consuming this type of beverages.

In addition to changes in eating habits, alcoholic drinks and ultra-processed food consumption was the lowest and showed no changes before and during social isolation. Some studies suggest that increased alcohol consumption during the COVID-19 pandemic may be a response to boredom, inactivity, isolation, and as a means of combating anxiety, stress, and/or loss of sleep as a result of containment measures introduced to minimize the spread of the virus [39].

Changes towards a better quality of eating habits are necessary for the Peruvian population, especially in the context of the COVID-19 pandemic, where Peru is the country with the most deaths per capita [40]. Recent studies show that a varied plant-based diet improves the immune response, can exert anti-inflammatory, antioxidant, cytoprotective, and even antiviral properties [41]. Additionally, the fibers found abundantly in fruit and vegetables improve the intestinal microbiota and provide protective phytochemicals [42]. Promoting the consumption of a healthy diet represents an important challenge for this country and could potentially prevent the future appearance of chronic diseases.

The main limitation of this study is the lack of representativeness of the population sample. Therefore, it is not possible to expand the results to the population of the whole country. There is also an overrepresentation of younger individuals (<25 years). Moreover, all data collected are self-reported, and this could make them not completely reliable, especially when it comes to reporting behaviors for which there may be a social stigma (e.g., alcohol). Another limitation could be the lack of information about religious practices, which could have influenced diet and lifestyle changes, as well as the lack of questions about whole food consumption. The findings are also limited by the absence of data on smoking and comorbidities, important factors affecting many lifestyles.

## 5. Conclusion

In conclusion, the findings of this study carried out in a Peruvian population during the COVID-19 pandemic demonstrated an increase in the practices of healthy eating habits, including a higher consumption of fruit, vegetables, legumes, and eggs and a lower intake of bakery products and ultra-processed foods. Also, more than 70% of the participants reported changes in their sleep pattern, where those who had been in social isolation for more than 61 days were 2.31 times as likely to experience sleep changes. Additionally, more than half indicated an increase in physical activity despite social isolation, where physical activity at home for at least 30 minutes per day and more days of practice per week significantly reduced the probability of high BMI. However, this study also showed that half of the participants reported weight gain. Future studies should consider additional potential factors, starting with the observation in this study that most participants had a changed sleep pattern during social isolation and potentially including other factors like stress, type of exercise, pedometer step counts, and portion sizes.

## Figures and Tables

**Table 1:** Sociodemographic and anthropometric data.

Variables	Total (*n* = 1176)	Men (*n* = 571)	Women (*n* = 605)	*p* value
Age				
18–25 yrs	522 (44.4)	197 (34.5)	325 (53.7)	<0.001^*∗*^
26–40 yrs	396 (33.7)	213 (37.3)	183 (30.2)	
41–55 yrs	200 (17.0)	115 (20.1)	85 (14.0)	
56–70 yrs	58 (4.9)	46 (8.1)	12 (2.0)	
Marital status				
Single	796 (67.7)	339 (59.4)	457 (75.5)	<0.001^*∗*^
Married	333 (28.3)	205 (35.9)	128 (21.2)	
Separated or divorced	42 (3.6)	25 (4.4)	17 (2.8)	
Widow(er)	5 (0.4)	2 (0.4)	3 (0.5)	
Level of education				
Primary incomplete	4 (0.3)	4 (0.7)	0 (0.0)	0.004^*∗*^
Primary completed	9 (0.8)	4 (0.7)	5 (0.8)	
Secondary completed	342 (29.1)	143 (25.0)	199 (32.9)	
Preparatory or tertiary completed	199 (16.9)	89 (15.6)	110 (18.2)	
University degree completed	447 (38.0)	237 (41.5)	210 (34.7)	
Graduate degree completed	175 (14.9)	94 (16.5)	81 (13.4)	
Occupational status				
Student	459 (39.0)	159 (27.8)	300 (49.6)	<0.001^*∗*^
Worker	441 (37.5)	272 (47.6)	169 (27.9)	
Worker and student	194 (16.5)	110 (19.3)	84 (13.9)	
Housewife	19 (1.6)	1 (0.2)	18 (3.0)	
Unemployed	58 (4.9)	25 (4.4)	33 (5.5)	
Retired	5 (0.4)	4 (0.7)	1 (0.2)	
Employment status				
I am not working	541 (46.0)	189 (33.1)	352 (58.2)	<0.001^*∗*^
Formal job with a contract	362 (30.8)	201 (35.2)	161 (26.6)	
Self-employed	159 (13.5)	114 (20.0)	45 (7.4)	
Public sector	114 (9.7)	67 (11.7)	47 (7.8)	
Days under social isolation				
Have not socially isolated	143 (12.2)	68 (11.9)	75 (12.4)	0.019^*∗*^
Less than 15 days	37 (3.1)	16 (2.8)	21 (3.5)	
15 to 30 days	59 (5.0)	36 (6.3)	23 (3.8)	
31 to 45 days	41 (3.5)	25 (4.4)	16 (2.6)	
45 to 60 days	101 (8.6)	60 (10.5)	41 (6.8)	
More than 61 days	795 (67.6)	366 (64.1)	429 (70.9)	
Body Mass Index (BMI)				
Malnourished	18 (1.5)	5 (0.9)	13 (2.1)	<0.001^*∗*^
Normal	639 (54.3)	245 (42.9)	394 (65.1)	
Overweight	420 (35.7)	258 (45.2)	162 (26.8)	
Obesity I	84 (7.1)	52 (9.1)	32 (5.3)	
Obesity II	15 (1.3)	11 (1.9)	4 (0.7)	
Weight change during social isolation (gained)				
1–3 kg	245 (20.8)	105 (18.4)	140 (23.1)	0.001^*∗*^
4–7 kg	161 (13.7)	87 (15.2)	74 (12.2)	
8–11 kg	41 (3.5)	28 (4.9)	13 (2.1)	
>12 kg	14 (1.2)	6 (1.1)	8 (1.3)	
Yes, but unsure how much	185 (15.7)	72 (12.6)	113 (18.7)	
No	530 (45.1)	273 (47.8)	257 (42.5)	
COVID-19 diagnosis confirmed				
No	1074 (91.3)	521 (91.2)	553 (91.4)	1
Yes	102 (8.7)	50 (8.8)	52 (8.6)	

Data expressed as absolute and relative frequency (%). COVID-19, coronavirus disease; yrs, years. ^*∗*^Statistically significant, *p* < 0.05.

**Table 2:** Lifestyles during social isolation.

Variables	Total (*n* = 1176)	Men (*n* = 571)	Women (*n* = 605)	*p* value
Physical activity (PA)				
No	532 (45.2)	258 (45.2)	274 (45.3)	1
Yes	644 (54.8)	313 (54.8)	331 (54.7)	
Days per week				
None	532 (45.2)	258 (45.2)	274 (45.3)	0.204
1 to 2 days	173 (14.7)	76 (13.3)	97 (16.0)	
3 to 4 days	310 (26.4)	147 (25.7)	163 (26.9)	
5 to 6 days	131 (11.1)	71 (12.4)	60 (9.9)	
7 days	30 (2.6)	19 (3.3)	11 (1.8)	
Duration				
No physical activity	532 (45.2)	258 (45.2)	274 (45.3)	0.425
Less than 30 minutes	257 (21.9)	122 (21.4)	135 (22.3)	
30 minutes to 1 hour	335 (28.5)	159 (27.8)	176 (29.1)	
1 to 2 hours	46 (3.9)	28 (4.9)	18 (3.0)	
Other	6 (0.5)	4 (0.7)	2 (0.3)	
Sleep routine				
No change	304 (25.9)	171 (29.9)	133 (22.0)	0.006^∗^
Sleep more	434 (36.9)	204 (35.7)	230 (38.0)	
Sleep less	438 (37.2)	196 (34.3)	242 (40.0)	
Hours of sleep				
0 to 3 hours	25 (2.1)	12 (2.1)	13 (2.1)	0.170
4 to 6 hours	419 (35.6)	188 (32.9)	231 (38.2)	
7 to 9 hours	651 (55.4)	335 (58.7)	316 (52.2)	
>10 hours	81 (6.9)	36 (6.3)	45 (7.4)	

Data expressed as absolute and relative frequency (%). ^∗^Statistically significant, *p* < 0.05.

**Table 3:** Changes in consumption of food before and during social isolation.

Type of food	Low^a^	High^a^	OR	(CI95%)	*p* value
Vegetables					
Before	628 (53.4)	548 (46.6)	1	(Reference)	<0.01
During	569 (48.4)	607 (51.6)	1.56	(1.21–2.00)	
Fruit					
Before	627 (53.3)	549 (46.7)	1	(Reference)	<0.01
During	579 (49.2)	597 (50.8)	1.42	(1.10–1.81)	
Legumes					
Before	927 (78.8)	249 (21.2)	1	(Reference)	<0.01
During	881 (74.9)	295 (25.1)	1.67	(1.23–2.28)	
Eggs					
Before	887 (75.4)	289 (24.6)	1	(Reference)	<0.01
During	808 (68.7)	368 (31.3)	2.00	(1.52–2.65)	
Bakery products					
Before	699 (59.4)	477 (40.6)	1	(Reference)	<0.05
During	733 (62.3)	443 (37.7)	0.74	(0.56–0.97)	

Data expressed as absolute and relative frequency (%). OR: odds ratios; CI95%, confidence interval 95%. ^a^Consumption of food.

**Table 4:** Factors associated with high body mass index during the COVID-19 pandemic in the study sample.

Variables	BMI		ORc (CI95%)	ORa (CI95%)
	<25 kg/m^2^ (*n* = 657)	≥25 kg/m^2^ (*n* = 519)		
Age (years)	28.0 ± 11.0	35.1 ± 12.4	1.05 (1.04–1.06)^*∗∗*^	—
Sex (%)				
Men	250 (43.8)	321 (56.2)	1	—
Women	407 (67.3)	198 (32.7)	0.38 (0.30–0.48)^*∗∗*^	—
Vegetables (%)				
No	299 (52.5)	270 (47.5)	1	1
Yes	358 (59.0)	249 (41.0)	0.77 (0.61–0.97)^*∗*^	0.87 (0.68–1.11)
PA at home (%)				
No	255 (47.9)	277 (52.1)	1	1
Yes	402 (62.4)	242 (37.6)	0.55 (0.44–0.70)^*∗∗*^	0.58 (0.45–0.74)^*∗∗*^
PA per week (days)	2.1 ± 2.1	1.6 ± 2.0	0.89 (0.84–0.94)^*∗∗*^	0.89 (0.84–0.94)^*∗∗*^
Time of PA (%)				
No physical activity	255 (47.9)	277 (52.1)	1	1
Less than 30 minutes	156 (60.7)	101 (39.3)	0.60 (0.44–0.81)^*∗∗*^	0.61 (0.44–0.84)^*∗∗*^
30 minutes to 1 hour	217 (64.8)	118 (35.2)	0.50 (0.38–0.66)^*∗∗*^	0.54 (0.40–0.73)^*∗∗*^
1 to 2 hours	27 (58.7)	19 (41.3)	0.65 (0.35–1.19)	0.64 (0.33–1.21)
Others	2 (33.3)	4 (66.7)	1.84 (0.36–13.36)	1.01 (0.18–8.03)

Data expressed as absolute and relative frequency (%) or mean ± SD. cOR, crude odds ratios; aOR, adjusted odds ratio by all sex and age; CI95%, confidence interval 95%; PA, physical activity. ^∗∗^*P* < 0.01.

**Table 5:** Factors associated with sleep changes during the COVID-19 pandemic in the study sample.

Variables	Sleep changes		ORc (IC95%)	ORa (IC95%)
	No (*n* = 304)	Yes (*n* = 872)		
Age (years)	33.5 ± 12.9	30.3 ± 11.8	0.98 (0.97–0.99)^*∗∗*^	—
Sex (%)				
Men	171 (29.9)	400 (70.1)	1	—
Women	133 (22.0)	472 (78.0)	1.52 (1.17–1.98)^*∗∗*^	—
Social isolation (%)				
Have not been socially isolated	54 (37.8)	89 (62.2)	1	1
Less than 15 days	15 (40.5)	22 (59.5)	0.89 (0.43–1.89)	0.84 (0.40–1.79)
15 to 30 days	17 (28.8)	42 (71.2)	1.50 (0.79–2.95)	1.65 (0.86–3.26)
31 to 45 days	19 (46.3)	22 (53.7)	0.70 (0.35–1.42)	0.78 (0.38–1.59)
45 to 60 days	33 (32.7)	68 (67.3)	1.25 (0.73–2.15)	1.35 (0.79–2.34)
More than 61 days	166 (20.9)	629 (79.1)	2.30 (1.57–3.35)^*∗∗*^	2.31 (1.57–3.38)^*∗∗*^
Meals per day	3.8 ± 1.1	4.1 ± 1.2	1.21 (1.08–1.35)^*∗∗*^	1.17 (1.05–1.32)^*∗∗*^

Data expressed as absolute and relative frequency (%) or mean ± SD. cOR, crude odds ratios; aOR, adjusted odds ratio by all sex and age; CI95%, confidence interval 95%; ^∗∗^*P* < 0.01.

## Data Availability

The data presented in this study are available on request from the corresponding author.
